# Coronary Smooth Muscle Cell Calcium Dynamics: Effects of Bifurcation Angle on Atheroprone Conditions

**DOI:** 10.3389/fphys.2018.01528

**Published:** 2018-10-31

**Authors:** Stewart Dowding, Constantine Zakkaroff, Stephen Moore, Tim David

**Affiliations:** ^1^UC High Performance Computing Centre, University of Canterbury, Christchurch, New Zealand; ^2^Department of Accounting and Information Systems, University of Canterbury, Christchurch, New Zealand; ^3^IBM Research, Melbourne, VIC, Australia

**Keywords:** coupled arterial cells, endothelial cells, smooth muscle cells, atherosclerosis, physiological modeling, parallel simulations, bifurcation angle, calcium dynamics

## Abstract

This work investigates the effect of arterial bifurcation angulation on atherosclerosis development through *in-silico* simulations of coupled cell dynamics. The computational model presented here combines cellular pathways, fluid dynamics, and physiologically-realistic vessel geometries as observed in the human vasculature. The coupled cells model includes endothelial cells (ECs) and smooth muscle cells (SMCs) with ion dynamics, hetero and homotypic coupling, as well as electro-diffusive coupling. Three arterial bifurcation surface models were used in the coupled cells simulations. All three simulations showed propagating waves of Ca^2+^ in both the SMC and EC layers, following the introduction of a luminal agonist, in this case ATP. Immediately following the introduction of ATP concentration Ca^2+^ waves propagate from the area of high ATP toward the areas of low ATP concentration, forming complex patterns where waves interact with eachother, collide and fade. These dynamic phenomena are repeated with a series of waves of slower velocity. The underlying motivation of this research was to examine the macro-scale phenomena, given that the characteristic length scales of atherosclerotic plaques are much larger than a single cell. The micro-scale dynamics were modeled on macro-scale arterial bifurcation surfaces containing over one million cells. The results of the simulations presented here suggest that susceptibility to atherosclerosis development depends on the bifurcation angulation. In conjunction with findings reported in the literature, the simulation results demonstrate that arterial bifurcations containing wider angles have a more prominent influence on the coupled cells pathways associated with the development of atherosclerosis, by means of disturbed flow and lower SMC Ca^2+^ concentrations. The discussion of the results considers the findings of this research within the context of the potential link between information transport through frequency encoding of Ca^2+^ wave dynamics and development of atheroprone conditions.

## 1. Introduction

Over the past 30 years, since the pioneering research of Caro et al. (1971), a substantial body of work has been carried out in order to elucidate the relationship between vascular geometry and the growth of atherosclerotic plaques. A recent review by Heo et al. (2014) shows the current views of flow-mediated influence on the development of atherosclerosis. Much has been discovered—however little has been attempted in order to explain the role of spatially varying agonists on cellular dynamics in combination with the cellular pathways located in the intima of the vessel wall, mediated by luminal agonists, and disturbed fluid flow.

### 1.1. Atherogenisis and vascular geometry

Multiple studies agree that disturbed vascular flow is the potential trigger for both endothelial and smooth muscle cell dysfunction in human vasculature, and lead to the activation of pro-inflammatory phenotype and coronary plaque formation (Libby, 2002; Davignon and Ganz, 2004; Plank et al., 2006). Similarly, a study by David (2003) pinpoints wall shear stress (WSS) and disturbed flow in wide-angle bifurcations as a mechanical level trigger of atherosclerosis. However, the underlying biochemical mechanisms of atherogenesis, potentially triggered by spatially varying agonist concentrations in the boundary layer flow remain unclear.

Further, it has been shown that the localization of plaque formation is associated with properties of arterial geometries especially in or close to the coronary and carotid bifurcations (Thomas et al., 2005; Lee et al., 2008; Dong et al., 2014). The review by Chiu and Chien (2011) demonstrates that the majority of plaque formation cases occur close to bifurcations and, more specifically on the outer walls of those bifurcations. Lee et al. (2008) have conducted a number of computational fluid dynamics (CFD) simulations on carotid bifurcation geometries derived from magnetic resonance imaging (MRI) data and found significant relationships between disturbed flow, proximal area ratio and bifurcation tortuosity, whilst Dong et al. (2014) concluded that wider bifurcation angles, specifically in the left circumflex (LCX) bifurcation shoulder, are likely sites of disturbed flow.

### 1.2. The frequency-mediated protein phosporylation hypothesis

In contrast to the view that [Ca^2+^] (calcium ion concentration) is the mediator for protein phosphorylation the work of Goldbeter et al. (1990) suggested that the [Ca^2+^] oscillatory frequency encodes for phosphorylation of proteins related to atherogenesis, where the phosphorylation kinetic rates were a function of [Ca^2+^] frequency. Hence one could hypothesize that the kinetic rates may be a marker for predisposition to kinase activation if the Ca^2+^ dynamics varied sufficiently in areas prone to plaque formation.

Multiple works indicate the possibility that cellular calcium dynamics, in terms of variations in frequency of oscillations, could enhance pathways that increase the probability of atherosclerotic plaque formation. For example, the mitogen-activated protein kinase (MAPK) pathway has been shown to be a crucial signaling mechanism in foam cell formation, endothelial cell (EC) activation, and vascular smooth muscle cell (SMC) proliferation (Hakala et al., 2006; Elliott and Eguchi, 2015). Our hypothesis is that Ca^2+^ dynamics could encode for MAPK pathway activation and that the resulting oscillatory [Ca^2+^] variations as a function of geometry may be a biomarker for the early initiation of plaque formation.

### 1.3. *In-silico* studies of Ca^2+^ dynamics

Variations in both blood flow, the associated wall shear stress and luminal surface agonist concentrations occur over length scales much larger than a single cell. A number of models were used to investigate the [Ca^2+^] dynamics phenomena occurring via coupled cells. However, none of them provided simulations which occur over the macro scale where characteristic lengths are centimeters rather than micrometers. Similarly, the studies reported to date included a limited number of pathways modeling hetero and homocellular communication. For example, Koenigsberger et al. (2006) modeled a vessel segment of 50 micrometers in length consisting of 80 SMCs and 30 ECs where coupling consisted of assuming a diffusive process for membrane potential. The experiments simulate pressure variation by including a stretch channel, but did not include any alternative pathways such as Nitric Oxide (NO). The work of Jacobsen et al. (2008) reported experiments with 45 coupled SMCs without an endothelial layer. The report by Koenigsberger et al. (2010) describes a model of a 2D tissue strip consisting of 130x3 SMCs.

A small number of studies report macro-scale simulations of arterial geometries which examine the coupling of intracellular chemical species. For example, the work of Shaikh et al. (2012) used a massively parallel one-dimensional (1D) model of coupled cells to investigate the propagation of [Ca^2+^] waves with spatially varying agonist concentrations. The results demonstrated that by varying the coupling coefficients, significantly different wave propagation profiles were obtained including propagation upstream with respect to blood flow. Further, the work of Boileau et al. (2015) provided a computational framework of cellular dynamics over large vessel segments with an emphasis on realistic arterial geometries generated from patient data. However, this work did not include a model of the endothelium, nor that of the myoendothelial gap junctions linking SMCs and ECs, otherwise known as heterotypic coupling. The experiments by Boileau et al. (2015) modeled membrane potential using a linear gradient term, rather than modeling physiologically realistic coupling which includes both Fickian and electro-diffusive terms.

### 1.4. Coupled cells dynamics simulations in arterial bifurcations

The model presented here combines cellular chemical pathways, fluid dynamics, and physiologically realistic vessel geometries. The micro-scale dynamics, including chemical species concentrations within individual ECs and SMCs were modeled on arterial bifurcation surfaces containing over one million cells. The coupled cells model includes EC and SMC ion dynamics, hetero and homotypic coupling, as well as Fickian and electro-diffusive models. The underlying motivation of this research was to examine the macro-scale phenomena, given that atherosclerotic plaques have characteristic length scales much larger than a single cell in the arterial wall.

In addition, the inclusion of CFD-derived agonist concentrations as a function of vessel geometry provides a state-of-the-art investigation into the relationship between spatially varying agonist concentrations, ionic dynamics, and vessel geometry. The model and the *in-silico* experiments presented in this work offer the opportunity to further the understanding of the complex relationship between atherosclerosis and vessel geometry.

The remainder of this article discusses the enhancements to the coupled cells model and simulation framework in section 2, followed by the simulations results in section 3. Section 4 considers the findings of this research within the context of the potential link between information transport through frequency encoding of Ca^2+^ wave dynamics and development of atheroprone conditions.

## 2. Methods

The coupled cells experiments presented in this work bring together a series of methodological enhancements which embody a step change in the area of *in-silico* atheroprone conditions modeling. These enhancements include physiologically realistic arterial geometries, an IP_3_ pathway and electro-diffusive coupling in the coupled cells model. While the foundation of the experimental framework is provided in the report by Zakkaroff et al. (2016), this section focuses on the details of the set of bifurcating arterial segments and the corresponding agonist maps along with the refined coupled cells model.

### 2.1. Bifurcating arterial surfaces

**Figure 1:**
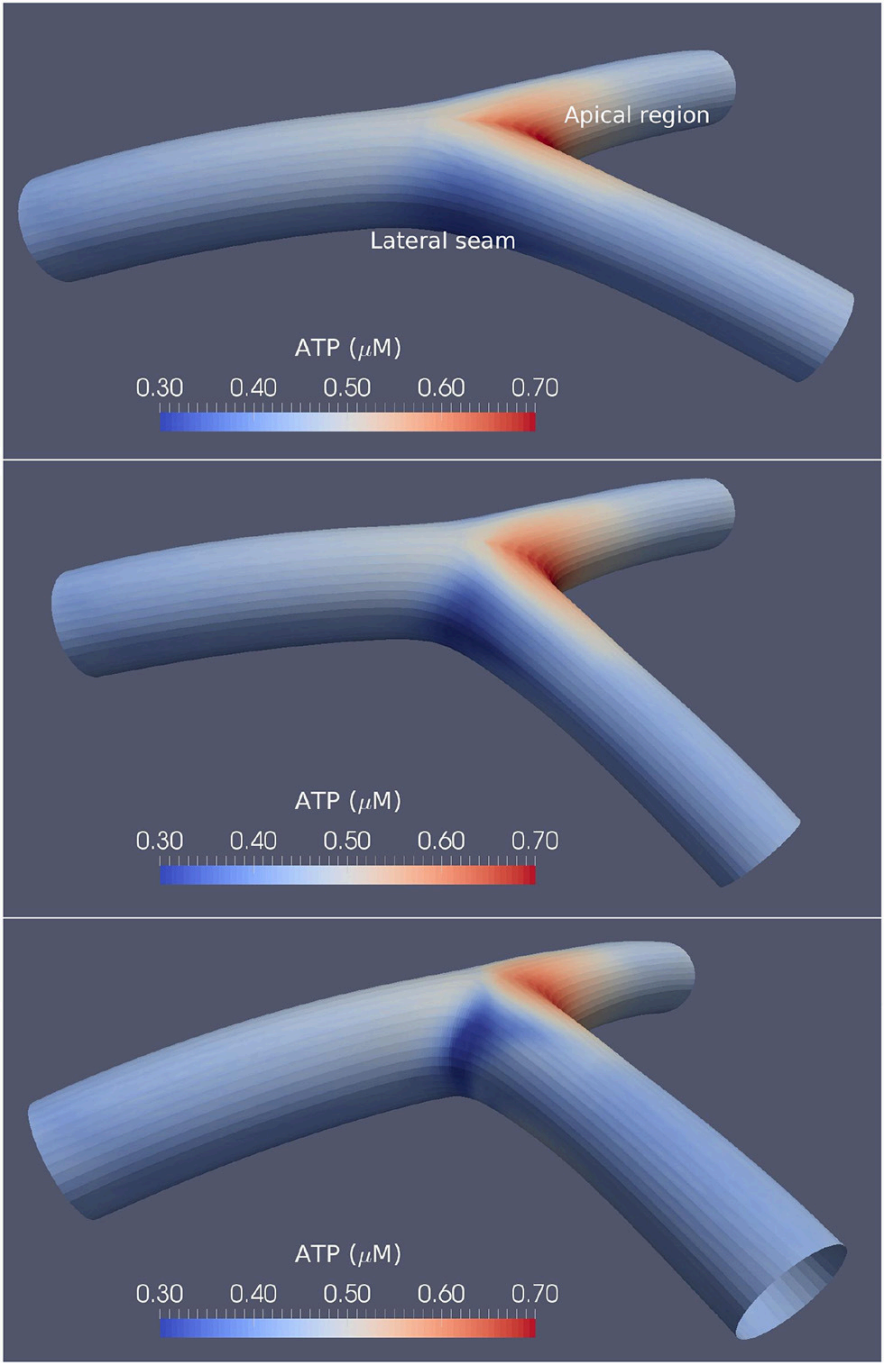
Arterial bifurcations and CFD-based ATP maps. The time-averaged ATP maps for the 50° **(Top)**, 80° **(Middle)**, and 110° **(Bottom)** arterial segments bifurcation generated using CFD. High ATP concentrations are observed at the apical regions, while the lateral seams of the arterial segments show low ATP concentrations. The large-angle bifurcations correspond to higher ATP concentrations in the areas of flow detachment over the bifurcation seams.

Three varying angles of bifurcation were chosen to represent the variation in human population: 50°, 80° and 110°, based on the physiological range of arterial bifurcation angle variation reported in the work of Dong et al. (2014). Figure 1 shows the three surface models along with the agonist map overlays, where each bifurcation model is approximately 18 mm in length and 2.5 mm in diameter. An arterial vessel surface of this scale may include over one million ECs and SMCs combined.

The method of creating EC and SMC surface meshes corresponding to endothelial and smooth muscle layers presented in Zakkaroff et al. (2016) was extended to enable the creation of physiologically accurate bifurcating arterial segments with varying angles between daughter branches.

In addition to the varying angles between branches, these surface meshes feature two additional geometric properties: first, they are non-planar—the vessels considered in this research belong to a coronary tree, which is naturally affected by curvature as the vessels wrap around the myocardial surface; second, the radii of the daughter branches are related by Murray's law:

(1) $$\begin{array}{l} r_{p}^{3}=r_{d_{1}}^{3}+r_{d_{2}}^{3}, \end{array}$$

which relates the radii of downstream branches to the main stem (Murray, 1926), where *r*_*p*_ is the radius of the parent branch while *r*_*d*_1__ and *r*_*d*_2__ are the radii of the respective daughter branches.

### 2.2. Agonist maps

Time-averaged agonist maps for the arterial bifurcation surfaces are shown in Figure 1. As the agonist, adenosine triphosphate (ATP), concentration was used as an input to the cellular model via the endothelium, special care was given to deriving a physiologically realistic spatial distribution for the concentration maps. Luminal receptor agonist concentration maps (concentration per unit area) were derived from steady-state CFD simulations as described in the work of Zakkaroff et al. (2016), where the surface ATP concentrations were derived from flowing ATP concentrations and a reactive state at the luminal wall. Due to the low diffusivity of agonists in flowing blood, time-dependent profiles have a negligible difference to that of steady state.

The agonist maps show relatively high ATP concentrations at the bifurcation apex, low concentrations at the lateral bifurcation seams, and near-uniform concentrations in all other regions. The work of Wan et al. (2008) provided ATP concentrations as a function of shear stress and found ATP concentrations between 0.2 and 1 μ*M*, while the results reported by Gorman et al. (2007) found the concentration of ATP in blood to be approximately 0.2 μ*M*. Variations of ATP in this research show similar concentrations to that of both Gorman et al. (2007) and Wan et al. (2008).

The agonist maps presented here link the geometric properties of a given arterial segment, via spatially varying WSS on the endothelium as shown by David (2003), with the cellular dynamics described by our physiological model. We note that while ATP is treated in this model as an example of an agonist, other mechano-transduction pathways exist, but they are not included in this model.

### 2.3. The coupled cells model

**Figure 2:**
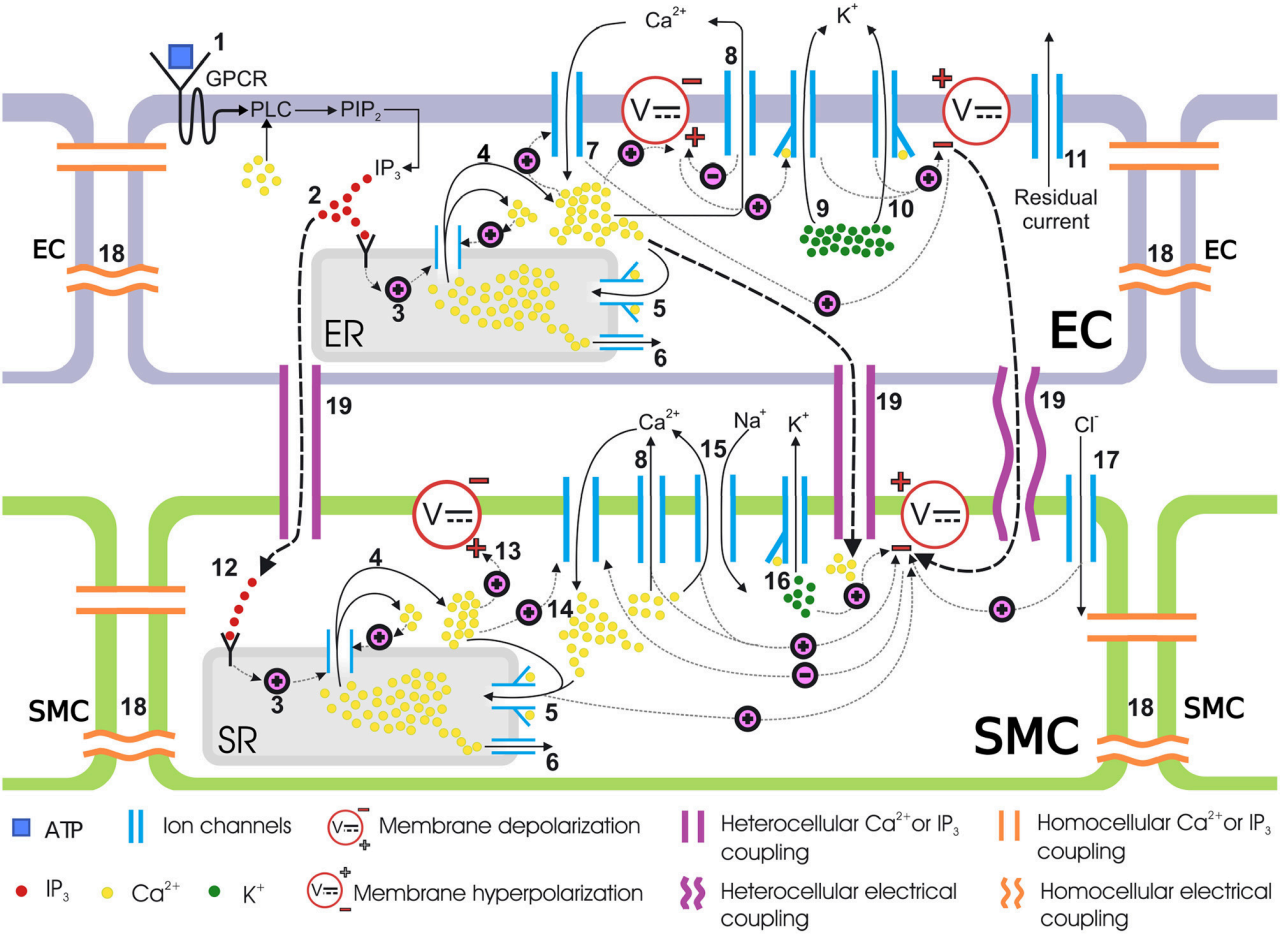
Coupled cells model. Schematic representation of mass transfer dynamics in a single coupled EC/SMC unit. (1) ATP binds to the purinergic P2Y receptors on the EC surface, activating the G-protein-coupled receptors (GPCR) which then activates a membrane-bound enzyme, phospholipase C (PLC), in the presence of Ca^2+^. PLC activation results in the phosphorylation of phosphatidylinositol 4,5-bisphosphate (PIP_2_). (2) PIP_2_ gives rise to IP_3_ which is then released in the cytosol. IP_3_ binds to IP_3_ receptors on the endoplasmic reticulum (ER)/sarcoplasmic reticulum (SR) surface. (3) IP_3_-bound IP_3_-receptor induces release of Ca^2+^ from the ER/SR into the cytosol. (4) Ca^2+^ release from intracellular store sensitizes the IP_3_-receptor further which releases more Ca^2+^, thus making a Ca^2+^ rich domain in the cytosol in both ECs and SMCs, which depolarizes the membrane potential. This is know as calcium-induced calcium release (CICR). (5) The sarco/endoplasmic reticulum Ca^2+^-ATPase (SERCA) pump requires ATP to move Ca^2+^ against the concentration gradient. Cytosolic Ca^2+^ encourages the replenishment of the intracellular stores via this pathway. (6) Ca^2+^ leaks from ER/SR consistently under a concentration gradient between cytosolic and ER/SR luminal Ca^2+^ and keeps the Ca^2+^ in equilibrium during a non-stimulated state of the cell. (7) The EC cytosolic Ca^2+^ favors the influx of extracellular Ca^2+^ from non-selective cation channels. (8) The SERCA pump pushes out cytosolic Ca^2+^ back into the SR. (9, 10) Activation of the KCa channel in the EC, upon binding to Ca^2+^ ions intracellularly at BKCa and SKCa, allows potassium ions (K^+^) to move out of the cytosol. This hyperpolarizes the membrane potential. (11) Residual current, predominantly consisting of monovalent ions, contributes to the membrane potential repolarization. (12) The IP_3_ concentration increases in the SMC cytosol via transmission of IP_3_ from coupled ECs. This IP_3_ increase activates the downstream IP_3_-induced Ca^2+^ release. (13) The IP_3_ induced and CICR Ca^2+^ depolarizes the membrane potential. (14) The membrane depolarization results in the influx of Ca^2+^ from voltage-operated calcium channels (VOCCs) which will close upon repolarization in the following steps. (15) Ca^2+^, in addition to other pathways, is pushed out via sodium ion Na^+^/Ca^2+^ exchanger. (16) Binding of Ca^2+^ ions to K_Ca_ opens BKCa channels in the SMC causing K^+^ ionic efflux and membrane repolarization. (17) Influx of chloride ions (Cl^−^) adds to the repolarization. (18) The medium for intercellular communication via homocellular gap junctions can either be Ca^2+^, IP_3_ or membrane potential gradients. (19) Heterocellular gap junctions can couple an EC to an SMC via Ca^2+^, IP_3_ or membrane potential coupling. Hyperpolarized EC membrane potential can hyperpolarize the SMC plasma membrane and consequently close VOCCs.

The coupled cells model presented in Zakkaroff et al. (2016) describes the binding of the agonist (ATP) to the luminal surface of the endothelium, which triggers a cascade of reactions and movements of ionic chemical species in both ECs and SMCs. Figure 2 provides an overview of the coupled cells model. However, the G-protein pathway responsible for IP_3_ generation via ATP binding to the luminal endothelial surface was reduced to a linear relationship between ATP and IP_3_ concentrations, which was a simplification in need of refinement. In contrast, this research considers the IP_3_ pathway in greater detail by building on the two models of Lemon et al. (2003) and Bennett et al. (2005).

#### 2.3.1. IP_3_ pathway

The IP_3_ dynamics in the coupled cells model used in this research, corresponding to the component 1 in Figure 2, were adapted from the work of Lemon et al. (2003) and are described as follows:

(2) $$\begin{array}{l} \frac{dĨ}{dt}=\epsilon \cdot r_{h}\cdot PIP_{2,tot}-J_{degrade}+\text{coupling terms}, \end{array}$$

where $\epsilon =u_{c}\cdot {\left(N_{a}\cdot V_{EC}\right)}^{-1}$ is a constant to ensure dimensional equivalence; ${PIP_{2}}_{tot}=5.0\times 10^{7}$ which is used in place of the variable PIP_2_ concentration, which typically shows little variance, as noted in the work of Johny et al. (2017); the coupling terms are the hetero and homoocellular diffusion rates of IP_3_ between neighboring ECs and SMCs.

The hydrolysis rate of PIP_2_ induced by fully activated PLC was modeled as follows:

(3) $$\begin{array}{l} r_{h}=\alpha \left(\frac{\tilde{c}}{\tilde{c}+K_{Ca}}\right){\tilde{G}}_{prot}, \end{array}$$

where *K*_*Ca*_ is the dissociation constant for Ca^2+^ binding to PLC, $\tilde{c}$ is the cytosolic concentration of Ca^2+^ in the EC, and α is an effective signal gain parameter which was fitted to experimental data, and is treated as a constant.

The dynamics of activated G-protein are described as:

(4) $$\begin{array}{l} \frac{d{\tilde{G}}_{prot}}{dt}=k_{a}\cdot \left(\delta +\rho_{P2Y}\right)\cdot \left(G_{prot_{tot}}-\tilde{G_{prot}}\right)-k_{d}\cdot {\tilde{G}}_{prot}, \end{array}$$

where *k*_*a*_ and *k*_*d*_ are the activation and deactivation rates of G-protein respectively; *G*_*pro*_*t*__*tot*__ is the total number of G-protein molecules within the EC; and δ is the G-protein intrinsic activity parameter. Finally, the ratio of bound to total P2Y receptors (under the assumption of fast kinetics), taken from Bennett et al. (2005), is as follows:

(5) $$\begin{array}{l} \rho_{P2Y}=\frac{\left[ATP\right]}{K_{ATP}+\left[ATP\right]}, \end{array}$$

where *K*_*ATP*_ is the effective Michaelis-Menten constant for ATP binding to a P2Y receptor and [*ATP*] is the lumenal ATP concentration. Complete definitions of the ODEs and fluxes in our model, including parameter values, are provided in the Supplementary Material.

#### 2.3.2. Electro-diffusive coupling

The work of Koenigsberger et al. (2005) offered a simplified hetero and homocellular coupling model based on an assumption that membrane potential is diffused between adjacent cells. A more accurate coupled cell model needs to consider that membrane potential is a phenomenon that exists due to the imbalance of charge between two separated bodies resulting in an electrical gradient, and hence cannot be diffused. Instead, ionic species carrying charge are diffused, which alters the membrane potential through electro-diffusion, in addition to concentration gradient-based Fickian diffusion.

Jacobsen et al. (2007) described the existence of gap junction currents explained by both membrane potential and concentration gradients between neighboring cells. This was modeled in the presented research as follows:

(6) $$\begin{array}{c} I_{gap,\phi }=P_{x}\sigma A_{segment}F\left[\nabla [\phi ]+\left(\frac{zF}{RT}\bar{\left[\phi \right]}\nabla V_{m}\right)\right]. \end{array}$$

Here $\bar{\left[\phi \right]}$ = ([ϕ]_*a*_+[ϕ]_*b*_)/2, which is the average concentration of ion ϕ between two cells *a* and *b*; $\nabla V_{m}≃\frac{V_{m,a}-V_{m,b}}{\delta x}$, which is the membrane potential gradient; $\nabla \left[\phi \right]≃\frac{{\left[\phi \right]}_{a}-{\left[\phi \right]}_{b}}{\delta x}$, which is the concentration gradient of ion ϕ; *A*_*segment*_ is the interface area of neighboring cells; and δ*x* is the distance between cells. The concentration gradient here is only relevant for Ca^2+^ in our model, as intracellular concentrations of all other ions were assumed to remain constant on the considered time scale. The values of these constant concentrations are taken from Kapela et al. (2008). The ion ϕ has the valence *z*; *F* is Faraday's Constant; *T* is the absolute temperature; *P*_*x*_ is the permeability of ion ϕ; and *R* is the universal gas constant. The currents described by Equation (6) were added to the hetero and homocellular coupling terms for membrane potential. The definitions, values, and dimensions of these parameters are provided in the Supplementary Material. IP_3_ coupling due to its zero valence is coupled using Fickian diffusion.

### 2.4. Coupling coefficients

The macroscopic mean conductance G in membrane potential coupling has been estimated by Van Rijen et al. (1997) to be 30 nS, whereas Lidington et al. (2000) estimated the macroscopic homocellular electrical resistance in ECs to be 3 MΩ (or $G=\frac{1}{3M\Omega }=333nS$). As $g=\frac{G}{C_{m}}$, the results of Van Rijen et al. (1997) yield a conductance one order of magnitude less than achieved by the evaluation of G from Lidington et al. (2000). Therefore, in the interest of coherent parameter use, the value of G = 30 nS was used here, as chosen in the work of Koenigsberger et al. (2005).

The membrane capacitance value between ECs is of the order of 30 pF as given by Schuster et al. (2003) and for SMCs it is 10 pF from Parthimos et al. (1999). Thus, for homocellular electrical coupling between SMCs, Yamamoto et al. (2001) calculated the macroscopic gap junctional resistance to be of the order of 90 MΩ (or $G=\frac{1}{90M\Omega }=11nS$). Therefore, the value of homocellular electrical coupling coefficients g and $\tilde{g}$ used for SMC and EC respectively, was 1000 s^−1^.

Experimental reports on the single channel conductance values of Cx40 and Cx43 gap junctions is scarce, and to the best of our knowledge the data on a cell's macroscopic or net conductance of intercellular Ca^2+^ and IP_3_ transfer is not available. The work of Koenigsberger et al. (2006) treat these values as free parameters and set the coefficients such that homocellular Ca^2+^ coupling between SMCs is able to synchronize the Ca^2+^ oscillations of five coupled SMCs in conjunction with the electrical coupling. SMCs are weakly coupled, and hence homocellular coupling coefficient was set to 0.05. EC homocellular Ca^2+^ coupling coefficient was also set to 0.05. In the case of heterocellular coupling, ECs and SMCs are coupled via the same three media.

Macroscopic intercellular resistance across myoendothelial junction has been estimated by Yamamoto et al. (2001) to be 900 MΩ, with the net capacitance between the two cells being approximately 20 pF, therefore making the heterocellular electrical coupling coefficient 50 s^−1^. For Ca^2+^ and IP_3_ transport across the myoendothelial junction, information on permeability is scarce and Koenigsberger et al. (2005) set their coupling coefficient to 0.05s^−1^ for IP_3_. The authors in Kapela et al. (2009) reduced the heterocellular IP_3_ coupling coefficient by assuming that myoendothelial coupling was weak, using a gap junction resistance of 13.5 GΩ, however this came from porcine coronary arteries. The variation in unitary conductance is large, judging from the current publications.

#### 2.4.1. NO, EDH, and EET considerations

Fast [Ca^2+^] changes in ECs and SMCs occur due to the exchange of Ca^2+^ in the ER/SR and the cytosol. On the one hand, the characteristic timescales for this are of the order of a second to several seconds. On the other hand, kinetic parameters that mediate cytosolic Ca^2+^ are of the order of 50 to 100 s, as noted in the work of Yang et al. (2005). We therefore note that the NO pathway is relatively slow and does not play an important part in wave propagation. Thus, although nitric oxide (NO) is a recognized antithrombic vasodilator, it is speculated here that due to the difference in the rates of reaction kinetics it does not influence the Ca^2+^ dynamics investigated in this work. A further examination of the NO-induced influences on cellular function other than cytosolic Ca^2+^ oscillations, is offered in section 4. In addition, it is recognized here that endothelial derived hyperpolarizing factor (EDH) may decrease Ca^2+^ levels, but experimental evidence on this pathway is still controversial.

The work of Hadfield et al. (2013) showed that epoxyeicosatrienoic acid (EET) production follows that of Ca^2+^. If one assumes that a similar pathway exists in the EC and SMC then the EET dynamics would induce potassium efflux, close the VOCC and thence reduce [Ca^2+^] with a possible reduction in [Ca^2+^] oscillations. Potentially, this may have the effect of inhibiting the MAPK pathway, and possibly inhibiting the initiation of plaque growth. However, the model of Hadfield et al. (2013) did not include a VOCC. The model presented in this research does not include an EET influence and it is still unknown as to the nominal value of EET produced in the coronary vasculature. Similarly, it is still unclear exactly how EET influences the BK channel. Thus, the role of EET in the Ca^2+^ dynamics is left out of this work, as it has yet to be fully investigated in the further work.

### 2.5. Parallel implementation

To enable parallel simulations of the coupled cells dynamics the arterial bifurcation surfaces were subdivided into quadrilateral domains. The quadrilaterals were grouped into three 2D surface-meshes, each representing a tubular segment of a bifurcation. Within each segment, the quadrilaterals corresponded to computational domains that were mapped to distinct tasks/cores in the Message Passing Interface (MPI)-enabled parallel environment. A periodic boundary condition was imposed along the longitudinal edge of each 2D grid. In this case, the periodic boundary condition refers to the communication-specific mapping of MPI tasks/domains. Each domain included 208 ECs and 80 SMCs, coming to 288 cells per quadrilateral, with a total of 1,175 million cells, spread across 4080 quadrilateral domains. The parallel implementation of the coupled cells simulations presented here is based on the work of Zakkaroff et al. (2016).

The system of ODEs simulating the time-dependent transfer of ions and membrane potential within each quadrilateral domain was solved at a specified time-step. For the simulations presented here the inter-domain communication interval was set to 0.01 s. This interval was chosen on the basis of a number of tests to maintain the balance between MPI communication and computation times. At regular time intervals the state variables from the cells along the edges of the quadrilateral domains were passed to the adjacent cells in the neighboring domains. The inter-domain communication was implemented through the MPI library, the solution of which follows the mesh ghost cell communication pattern recommended for discretised domain decomposition (Gropp et al., 2014). The solution for the ODEs was obtained by using the numeric differential equation solver ARKode, from the Suite of Nonlinear and Differential/Algebraic Equation Solvers (SUNDIALS) (Hindmarsh et al., 2005).

The simulations were performed on an IBM POWER7 architecture, each simulation using 120 cores, with each core solving 34 quadrilateral domains. Approximately 20 h of wall clock time was required to complete each of the 500-physiological-second simulations. Values of state variables, such as Ca^2+^ concentrations and membrane potential, for all ECs and SMCs were recorded after every elapsed physiological second. During post-processing, the output data were mapped onto the surface meshes and visualized and analyzed in ParaView (Ayachit, 2015).

## 3. Results

**Figure 3:**
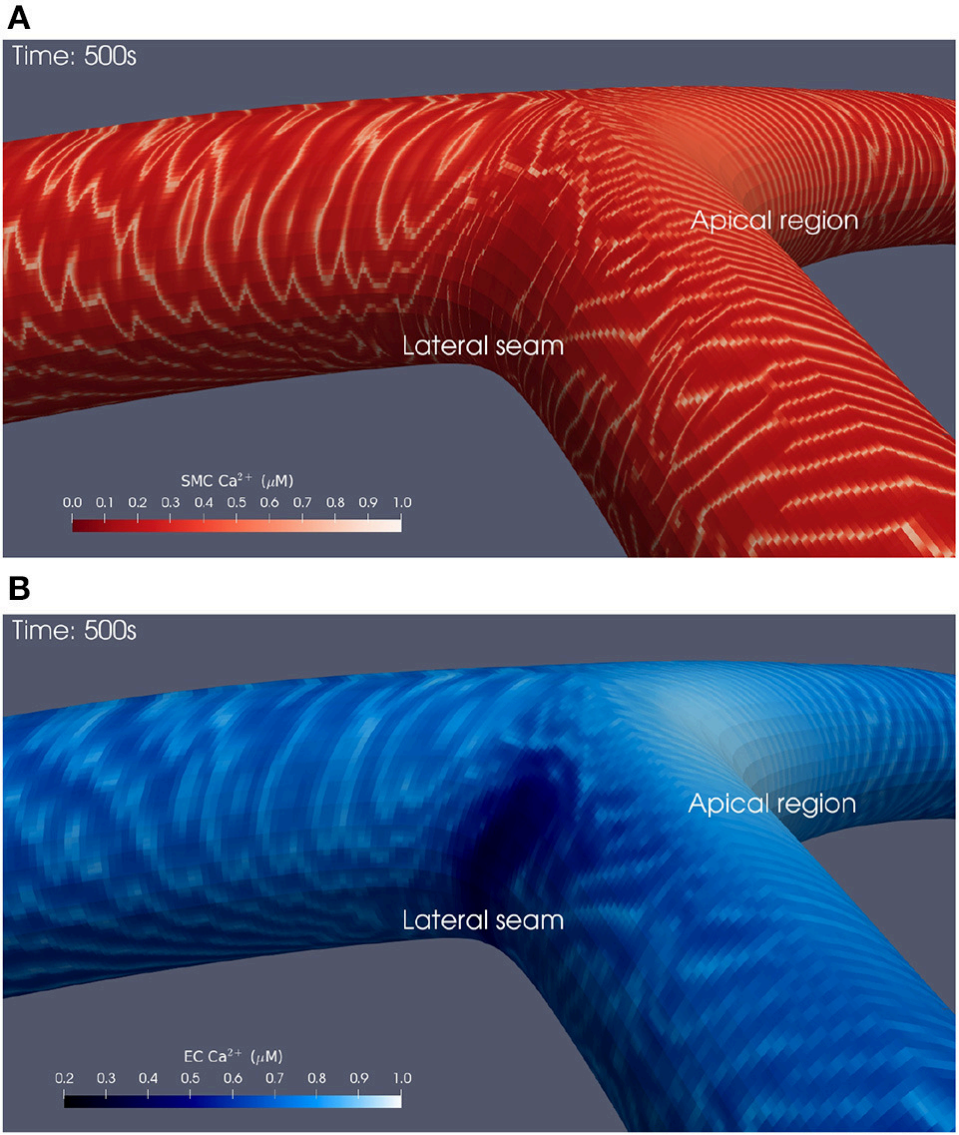
Simulation at t = 500 physiological seconds of Ca^2+^ dynamics in SMCs and ECs using a 110° geometry. In the SMCs **(A)**, waves of Ca^2+^ with maximal amplitude of 1 μM propagated outwards from the apical region. These waves struggled to penetrate into the lateral seams where the SMC Ca^2+^ concentration remained below 0.1 μM. Similar behavior was observed in the ECs **(B)**.

Snapshots of Ca^2+^ concentrations in SMCs and ECs for the bifurcation angle of 110° are presented in Figures 3A,B for *t* = 500 physiological seconds. The results demonstrated that ATP-induced production of IP_3_ in the endothelium and its subsequent diffusion into the SMC induced cytosolic Ca^2+^ oscillations for certain ATP concentrations, where the frequency of oscillation is a monotonic increasing function of the production rate of endothelial IP_3_. Given that IP_3_ is determined by the spatially varying ATP concentration at the endothelial wall, SMCs can oscillate as a function of space. However, a greater appreciation of the complexity of this phenomenon, observable in the form of slow-moving waves both up and downstream through the arterial wall, is gained by viewing the animated visualizations available on the University of Canterbury High Performance Computing YouTube channel^1^, which show cellular dynamics over the whole of each simulation.

### 3.1. Wave patterns

The waves of propagating SMC Ca^2+^ oscillations were observable in all simulations. Immediately following the introduction of the ATP concentration Ca^2+^ waves propagate from the area of high ATP concentration—the apex of the bifurcation. The initial wave front moves relatively quickly in a circumferential direction until it reaches the parent artery stem where it spreads out, reaching the upstream edge in less than 15 s. The wave moves to the outer wall where ATP concentration is low and forms a circular pattern, reducing in radius as time progresses. These dynamic phenomena are repeated with a series of waves slower in velocity. The period of the oscillations emanating from the apex of the bifurcation was approximately 20 s.

As time increases the uppermost parts of the circular waves in the outer wall become unstable and form complex patterns within areas of low ATP concentration, similar to the phenomenon of re-entrant waves. At approximately 350 physiological seconds into the simulation the low ATP concentration area has become highly complex as smooth Ca^2+^ waves move downstream along the inner wall of the daughter arteries, and the outer walls produce elaborate wave structures similar to that found in low ATP concentration regions. Waves also move upstream toward the inlet with a leading edge positioned at the top of the artery.

The Ca^2+^ waves produced at the apex were rapid and distinct, while those at the lateral bifurcation seams propagated more slowly, sporadically, and over significantly shorter distances. Larger bifurcation angles appeared to accentuate these observations, most notably the Ca^2+^ dynamics at the lateral seams of the 110° bifurcation geometry. Head-on collisions of Ca^2+^ waves result in the destruction of both waves.

Finally, the area affected by this complex Ca^2+^ behavior reduced in size as a function of wider bifurcation angles, seemingly as the low ATP concentration area (lateral seam as shown in Figure 1) reduces in size as the angulation increases. In both SMCs and ECs [Ca^2+^] oscillates with a slight phase lag.

### 3.2. Temporal averaging and surface mesh unwrapping

There exist significant differences in time-scales between the cardiac pulse, Ca^2+^ wave frequency, and atherosclerotic plaque formation. The temporal oscillatory behavior of EC and SMC Ca^2+^ concentrations and their influence on protein phosphorylation is complex and it is somewhat difficult, if not impossible, to describe it quantitatively in the absence of dynamic visualizations. Temporal averaging was used with the view of examining the influence of the dynamic behavior in the areas of high and low ATP concentration on MAPK pathway. Temporal averaging was applied for each cell *x* in the surface arterial mesh as follows:

(7) $$\begin{array}{l} {\left[{\bar{Ca}}^{2+}\right]}_{x}=\frac{1}{\left(t_{f}-t_{i}\right)}\sum_{t=t_{i}}^{t_{f}}{\left[Ca^{2+}\right]}_{x}\left(t\right). \end{array}$$

**Figure 4:**
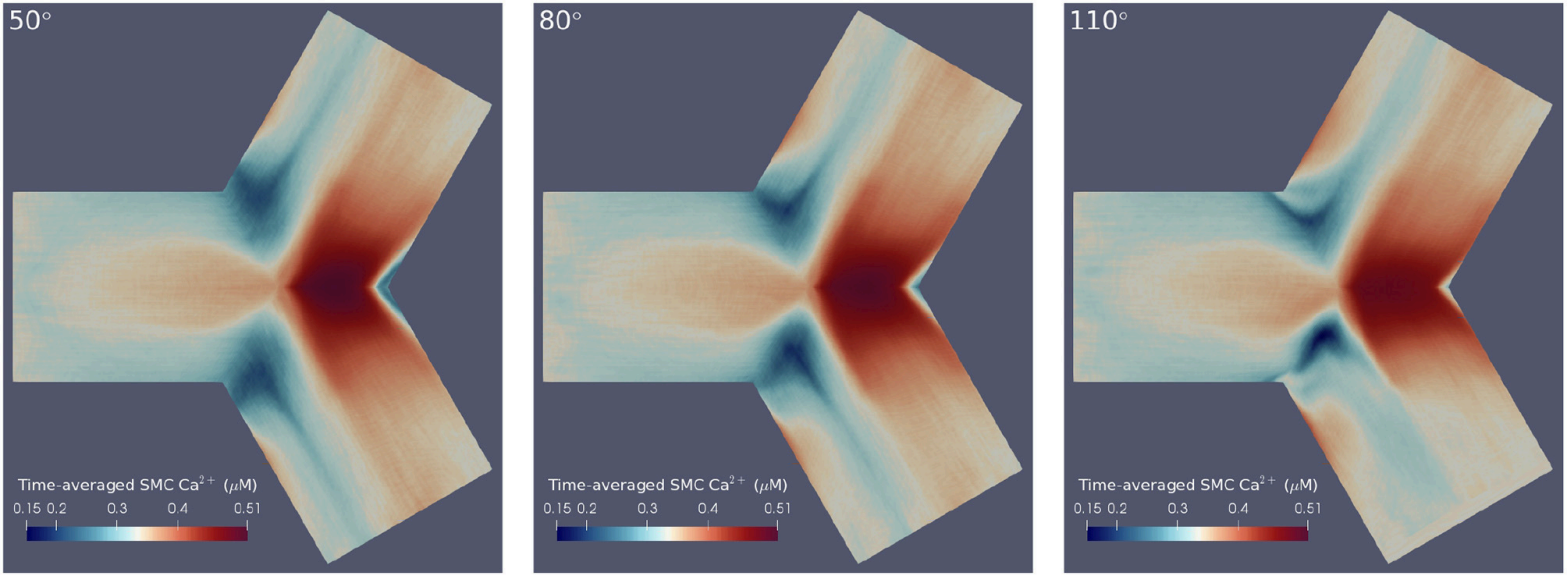
Time-averaged SMC Ca^2+^ concentration over 200 physiological seconds using flat mesh re-mappings of 50° **(Left)**, 80° **(Center)**, and 110° **(Right)** bifurcation geometries. We observed high averaged Ca^2+^ concentrations at the apical regions and low averaged Ca^2+^ concentrations at the lateral seams in all three geometries. Further, greater disturbances in Ca^2+^ wave propagation occurred at the lateral seams of geometries with wider bifurcation angles.

Here ${\left[{\bar{Ca}}^{2+}\right]}_{x}$ is the averaged Ca^2+^ concentration in cell *x* between times *t*_*i*_ and *t*_*f*_, and ${\left[Ca^{2+}\right]}_{x}\left(t\right)$ is the Ca^2+^ concentration in cell *x* at time *t*. Thus, the maximum/minimum values of oscillations were averaged to provide a more interpretable account of cellular dynamics. For ease of comparison, the differently angled bifurcations were mapped to a 2D surface, to allow a single perspective to capture [Ca^2+^] dynamics over the entire arterial segment. The circumferential edges on these surfaces are periodic and thus, this re-mapping can be thought of as an unwrapping of the original bifurcations. The result of applying these post-processing techniques is shown in Figure 4 for SMC Ca^2+^ values. Temporal averaging was performed over the final 200 s of the simulation such that *t*_*i*_ = 300 s and *t*_*f*_ = 500 s. These values were chosen in order to exclude transient behavior, while averaging Ca^2+^ concentrations over a sufficiently long period.

The results confirmed the observation that greater disturbances in Ca^2+^ wave propagation occurred at the lateral seams of bifurcations with increasing bifurcation angle. Further, this temporal averaging disclosed that geometries with larger bifurcation angles were subject to lower Ca^2+^ concentrations at the bifurcation apex. The areas of averaged Ca^2+^ reduce in size as a function of increasing bifurcation angle. This is primarily due to the influence of fluid dynamics with the growing intensity of the rotational flow caused by the increase in curvature of the bifurcation Comerford et al. (2008) especially in the lateral region.

### 3.3. Protein phosphorylation

Our hypothesis has been, on the basis of Goldbeter's work, that frquency encoding in the form of [Ca^2+^] oscillations caused by cellular dynamics, mediated by geometry and spatial profile of the agonist, may influence the MAPK pathway, and hence induce plaque formation. Using the same notation as Goldbeter et al. (1990), the rate of change of the fraction of phosporylated protein, W^*^, can be written as:

(8) $$\begin{array}{l} \frac{dW^{*}}{dt}=\frac{v_{p}}{W_{T}}\left[\frac{v_{k}}{v_{p}}\frac{1-W^{*}}{K_{1}+1-W^{*}}-\frac{W^{*}}{K_{2}+W^{*}}\right], \end{array}$$

where

(9) $$\begin{array}{l} v_{k}=V_{mk}\frac{Ca^{2+}\left(t\right)}{K_{a}+Ca^{2+}\left(t\right)} \end{array}$$

**Figure 5:**
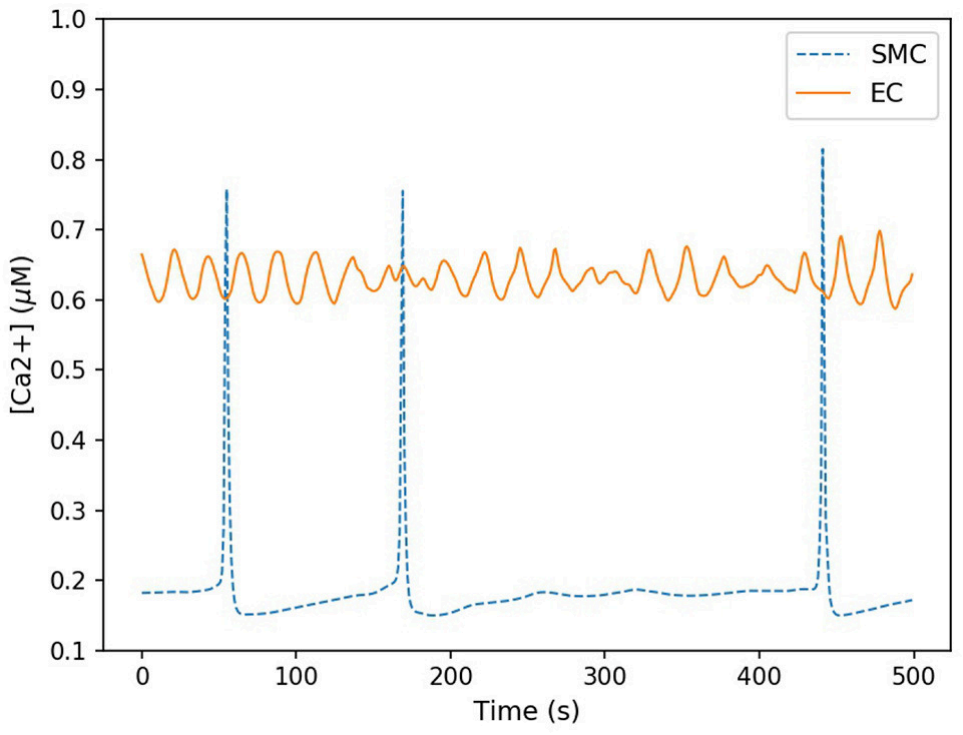
Time-dependent calcium profile for both SMC (blue) and EC (red) taken from the center of the lateral region. The SMC Ca^2+^ concentration spiked sporadicly, but for the majority of the time remained at a low concentration (approximately 0.2 μM). EC Ca^2+^ concentration oscillated within a small range approximately between 0.6 and 0.7 μM.

**Figure 6:**
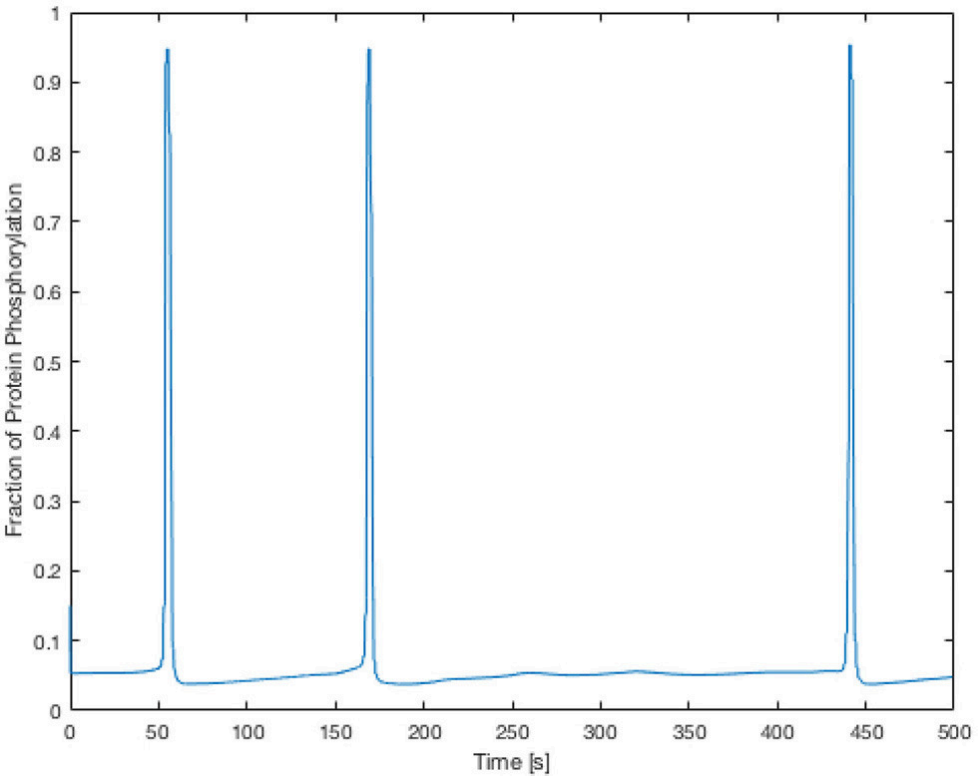
Time-dependent profile of the fraction of phosphorylated protein using the SMC Ca^2+^ profile shown in Figure 5. The phosphorylated protein dynamics follow the Ca^2+^ dynamics closely due to the relatively fast reaction rates.

Here the components in the brackets correspond to the production and decay of the phosphorylated protein respectively. Both of these reactions are assumed to be defined by Michaelis-Menten kinetics. The maximum rate of phosphorylated protein production is a function of the cellular cytosolic Ca^2+^ concentration. As [Ca^2+^] oscillates, production of W^*^ follows. The simulations of W^*^ in this work use the same parameter values as Goldbeter, and can be found in the Supplementary Material. Figure 5 shows the time-dependent Ca^2+^ profile for a single EC and SMC taken from the center of the low ATP concentration in the lateral region, whilst Figure 6 shows the resulting phosphorylated protein W^*^using the SMC Ca^2+^ taken from the profile shown in Figure 5.

The Ca^2+^ profile shows sporadic spiking with maximum amplitudes of up to 0.8 μM. However, most of the time the Ca^2+^ concentration is low: approximately 0.2 μM. Figure 6 shows that the phosphorylated protein dynamics follow the Ca^2+^ dynamics due to the relatively fast reaction rates, compared to the Ca^2+^ frequency, with maximum values of 0.95 μM. Most of time the phosphorylated protein concentration is low. On the basis of this result the lateral region, where one would expect plaque growth over time, the activation of MAPK pathway is low.

Since plaque formation is a chronic condition, and it may take months to form, a time-averaged value for the fraction of the phosphorylated protein is a more realistic parameter to investigate, which is defined as follows:

(10) $$\begin{array}{l} \bar{W^{*}}=\frac{1}{T_{max}-T_{min}}\overset{T_{max}}{\underset{T_{min}}{\int }}W^{*}\left(s\right)ds, \end{array}$$

where *T*_*min*_ is zero, and *T*_*max*_ to the time at the end of the simulation.

**Figure 7:**
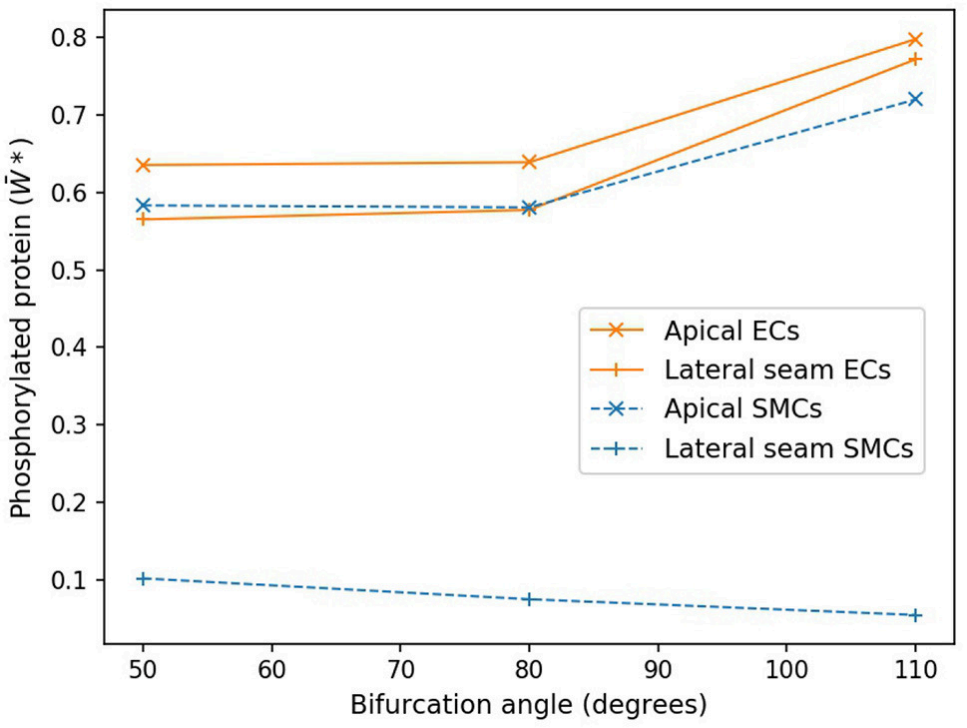
Protein phosphorylation for ECs and SMCs in geometries with varying angles. In the apical region, both the time averaged EC and SMC $\bar{W^{*}}$ values are increasing functions of bifurcation angle. Lateral seam ECs also follow this behavior, while lateral SMCs do not.

Figure 7 shows these average values as functions of cell type and region for the three angles of bifurcation. While in the apical region, both the time averaged EC and SMC $\bar{W^{*}}$values are increasing functions of bifurcation angle, that is not the case for the lateral region. Here EC-based $\bar{W^{*}}$increases with angle, but SMC-based $\bar{W^{*}}$ is a monotonic decreasing function and of much lower value. In the apical region the time-varying EC [Ca^2+^] values are near-constant and of relatively large value, of the order of 0.9 μM (data not shown), and the SMC [Ca^2+^] values are again near-constant, but lower, on average 0.4 μM (data not shown).

## 4. Discussion

All three simulations presented in this work showed propagating waves of Ca^2+^ in both the SMC and EC layers following the introduction of a luminal agonist, in this case ATP. The agonist binds to endothelial receptors, induces the production of IP_3_, and consequently the release of Ca^2+^ in both ECs and SMCs. Both ECs and SMCs are excitable and their Ca^2+^ concentrations can oscillate under certain conditions (Kapela et al., 2008). The work of Koenigsberger et al. (2005) used a strip of coupled ECs and SMCs to show that cells could develop in-phase oscillations, otherwise known as vasomotion, however those simulations did not include spatial agonist variation, nor the resultant wave propagation. Since the cells in the experiments presented in this work did not produce in-phase [Ca^2+^] variations, the phenomenon produced is not vasomotion.

Complex behavior in SMC Ca^2+^ dynamics was observed at the lateral bifurcation seams. In particular, these areas exhibited relatively higher Ca^2+^ concentrations in ECs, while Ca^2+^ waves in SMCs propagated in a slower, non-uniform manner, and over significantly shorter distances. These sporadic Ca^2+^ oscillations in the SMC seem to be primarily due to the cell being in or close to a steady state and receiving a diffusive flux of Ca^2+^ from the EC via gap junctions, which perturbs them into an oscillatory state and, as a consequence, provides sufficient cytosolic Ca^2+^ to excite the cells into continuing the propagating wave. In agreement with previous simulations presented in the work of Zakkaroff et al. (2016), the Ca^2+^ waves propagate through regions that are otherwise devoid of oscillations.

Pinto and Campos (2016) suggested that areas under arterial bifurcations are susceptible to potential plaque development, due to observed low time-averaged wall shear stress (TAWSS) and high relative resident time (RRT) values. Dong et al. (2014) presented a more specific hypothesis—that it is these areas under bifurcations with wider angles that are at greater risk of plaque formation. We observed low time-averaged Ca^2+^ concentrations in SMCs, in regions that CFD experiments have identified as sites most likely to experience plaque development (Steinman et al., 2002; Peiffer et al., 2013; Pinto and Campos, 2016) but contrastingly not for ECs. Further, we note the low Ca^2+^ concentrations in these areas are more prominent in arterial geometries with wider bifurcation angles, in agreement with the findings of Dong et al. (2014).

### 4.1. NO production and atheroprone conditions considerations

There is considerable evidence that the production of NO is reduced in areas of low WSS (Kanai et al., 1995; Malek et al., 1999; Kavdia and Popel, 2003), which in turn can lead to endothelial dysfunction (Davignon and Ganz, 2004) and may result in an increased probability of atherogenesis. The eNOS-mediated production of NO is a function of cytosolic Ca^2+^ concentration. Work by Comerford et al. (2008) showed that areas of eNOS reduced as the angle of the bifurcation increased. However, in terms of Ca^2+^ oscillations nitric oxide has no influence on the oscillatory dynamics, since this is primarily driven by the relative fluxes of the CICR mechanism and the SERCA pump associated with the SR storage of Ca^2+^.

Although the NO pathway does not appear in the presented model, we can rely on the eNOS and NO data from previous work. For example, the work of Dormanns et al. (2016) utilized the same model for eNOS generation as described by Comerford et al. (2008); their data indicated that the EC and SMC concentrations of NO, as determined from the eNOS concentration, were similar in value. Thus, we can surmise that even for conditions of oscillating Ca^2+^ concentration, areas of low ATP and time-averaged cytosolic EC and SMC Ca^2+^ concentration would also produce low areas of NO concentration, thereby possibly enhancing the progression of EC dysfunction and subsequent atherogenesis.

On the basis of a coupled cellular model combined with fluid dynamics, the areas of low WSS, ATP, and low NO seem to correlate with areas susceptible to plaque formation. At the same time the simulations reported here indicate that time averaged EC [Ca^2+^] is considerably higher than that of SMCs in the lateral region, giving rise to NO concentrations, compatable with the apical region where, from experiment, the probablity of plaque growth is low. This conflicting result warrants further investigation, and hence the inclusion of the full NO pathway along with WSS-mediated NO production.

### 4.2. Myoendothelial gap junctions, Cx43, and WSS

It has been suggested that geometric and subsequent biochemical changes are related to atherosclerotic regions (Caro et al., 1971; Ku et al., 1985). Ca^2+^ ions move between ECs and SMCs in healthy cellular states, primarily through the Cx43 connexin protein subunit. A concentration change in one layer will therefore affect the other, and so Ca^2+^ dynamics in both ECs and SMCs should be considered when investigating the onset of vascular disease.

The review of cell coupling and atherosclerosis by Burnier et al. (2009) states that “lesions are typically observed in arterial vessels with high WSS such as bifurcations,” in contrast to most accepted theories that low WSS is associated with atherosclerosis. The review, however, also states that the Cx43 gap junction is thought to be sensitive to mechanic stimuli due to its overexpression during shear stress, while there is evidence suggesting that this particular gap junction has increased expression in the intima during the early phases of atherosclerosis. The work of Inai et al. (2004) provides some evidence that Cx43 is increased in areas of high WSS gradients, although this was only expressed in ECs of cardiac valves. At the same time the work of DePaola et al. (1999) showed that Cx43 expression increased with increasing WSS, as well as disorganization of the Cx43 protein.

### 4.3. Frequency encoding and Ca^2+^ dynamics information transport

Meyer and Stryer (1988) suggested that calcium-activated proteins, such as calmodulin, are highly suited for detecting spikes in ionic concentrations, where calcium could excite different effector systems. First Goldbeter and Koshland (1981), and later Goldbeter et al. (1990) proposed the idea that [Ca^2+^] oscillations might be encoded in terms of their frequency. They suggested that one possible scenario of information transport through frequency encoding may be based on protein phosphorylation, but only in the case of zero-order kinetics, where the substrate concentration is far in excess. This provides support to the premise that protein phosphorylation is highly sensitive to changes in the kinetics. Goldbeter's analysis indicates that the time variation of the phosphorylated protein is directly associated with the oscillations of cytosolic Ca^2+^ and to the subsequent variation in kinase activity, where a higher frequency of the SMC Ca^2+^ oscillation produces a higher mean value of the phosphorylated protein.

Research by Goldbeter and Koshland (1981) and Goldbeter et al. (1990) further state that when “the period of oscillations is longer, the protein undergoes significant dephosphorylation from one Ca^2+^ peak to the next”, resulting in larger values of dephosphorylated protein. The results presented here indicate that along the outer wall of the bifurcations in areas of low ATP, where cytosolic Ca^2+^ oscillations are uncoordinated, the encoding information is disrupted causing possible dysfunction to those protein pathways. For example, as noted before MAPK pathways have been implicated in foam cell formation, EC activation, and vascular smooth muscle migration and proliferation, and several receptor tyrosine kinases (RTKs) pathways in a variety of cells have been shown to be important for the development of atherosclerosis.

It emerges that no single marker exists for plaque formation and that a number of conditions have to be present in order to initiate atherosclerotic conditions. On the basis of Ca^2+^ dynamics alone, aligned with variation in geometry and luminal wall agonist concentration, we conclude that initiation of atherosclerotic plaque may *not* be due to frequency encoded Ca^2+^ phosphorylation of the protein alone. However, combinations of more complex cellular models and the introduction of other pathways such as NO, along with the use of numerical simulations could provide further insight into the relationship of vascular geometry and atherosclerotic plaque formation.

## 5. Conclusions

This research examined single-cell dynamics on a macro-scale, and the presented model was compared with other mathematical models of cellular physiology in the related literature. The underlying coupled cells model was extended to include a detailed IP_3_ mediated Ca^2+^ pathway, and gap junction currents that replace simple linear gradients for membrane potential and Fickian diffusion of ions. The premise of this work was such that variations in the frequency and dynamics of the Ca^2+^ waves in areas of low WSS have the potential to influence the kinase reactions of proteins under the assumption that Ca^2+^ encodes for high sensitivity phosphorylation and possibly provides a marker for atherogenesis.

The macro-scale simulations in this work demonstrated oscillatory behavior in SMC and EC species' concentrations. Moreover, significantly lower Ca^2+^ concentrations were observed in areas under the lateral surface of bifurcations, in SMCs. The SMC Ca^2+^ dynamics in these areas were difficult to analyse at any given time point: waves propagated in a slow non-uniform manner, and over significantly shorter distances. Post-processing, in the form of time-averaging, was applied to gain insight into the emergent properties of these dynamics. This showed significantly lower Ca^2+^ concentrations over time at the lateral bifurcation seams compared to the other surface regions of the arterial segments. Ca^2+^ concentration frequencies and average values in the lateral domain for SMCs were relatively small. In contrast EC Ca^2+^ dynamics showed that average values were high in both apical and lateral regions. Since experiments have shown that plaque growth is rare in the apical region this would indicate that frequency encoding would not be a prominent mechanism in the very early stages of plaque growth.

In conjunction with findings reported in the literature, the results of this work suggest that arterial bifurcations containing wider angles may indeed influence the development of atherosclerosis, by means of disturbed flow and, consequently, lower SMC Ca^2+^ concentrations. The overal outcome of this work suggests that although Ca^2+^ dynamics are of considerable importance in cellular functions, other related pathways may combine in a non-linear fashion to produce an environment conducive to plaque formation.

This research provides a flexible simulation platform that allows for the investigation of cellular dynamics with geometrically varying, multi-scale arterial sections. The potential influence of bifurcation angulation on atherogenesis has been studied, and the investigation of many other phenomena is now possible. Finally, all additions and modifications made to this platform were designed and implemented to promote scalability and further model development. Future simulations of arterial physiology, including further detailed cellular chemistry and surface-meshes that span the entire coronary tree, should benefit from these extensions.

## 6. Limitations and future work

It is important to note that biophysical values and coefficients used in the presented model were collected from different configurations of experiments. For example, the coefficients of electro-diffusive coupling can vary widely depending on experimental setup. These considerations present a potential limitation for *in-silico* experiments; however, we hope that future research in physiology may help improve the homogenization of experimental findings for computational modeling.

For a given mesh, the ECs and SMCs on each quadrilateral were aligned orthogonally with respect to each other, where ECs align axially with the flow and the SMCs align circumferentially. These arrangements were generated such that they communicate with four neighboring cells homocellularly, when in fact an EC and SMC can typically communicate with up to six neighboring ECs and SMCs, respectively (Koenigsberger et al., 2005). Additionally, ECs tend to align in the direction of the dominant local WSS vector component whereas in the present case ECs are always aligned on an axial direction. The random nature of alignment for ECs at positions of low WSS has the potential to affect the homotypic and, possibly, heterotypic coupling coefficients as shown by Chiu and Chien (2011).

The NO pathway, including cyclic guanosine monophosphate (cGMP) in the EC, was not included in this model. This pathway describes the production of NO as a result of agonistic molecules within the lumen binding to EC receptors. NO has known atheroprotective properties, such as inhibiting the production of adhesion molecules that encourage the recruitment of monocytes. Following the implementation of this pathway in macro-scale simulations, it will be possible to examine NO dynamics across large sections of ECs, and, possibly, provide further insight into the process of atherosclerotic plaque development. Similarly, the introduction of stretch-activated channels and vasculature motion in the model may improve the fidelity of future simulation results.

Many geometric variations were considered in a study conducted by Lee et al. (2008), among which was bifurcation angulation. This property was chosen for analysis as a result of findings that bifurcations with wider angles are more likely to experience greater flow detachment (Dong et al., 2014). However, there are many other geometric properties (such as bifurcation tortuosity, for example) that are worthy of future exploration.

Shaikh et al. (2012) introduced multiple coupling cases to earlier Ca^2+^ dynamics simulations in order to model both healthy and pathological conditions in coupled cells behavior. These coupling cases were not the focus of the research presented here—the particular coupling cases modeling unhealthy cellular behavior already assume mid-to-late stages of atheroma development. However, future work should include the creation of new coupling cases that reflect initial stages of disease, before plaque formation is observable. Further, these coupling cases should vary both temporally and spatially as noted above due to the variation in EC alignment. Similarly, in their discussion of Ca^2+^ dynamics in the vascular endothelium, Taylor and Francis (2014) stated that it was important for future studies to move away from assumptions based on global Ca^2+^ changes and instead focus on spatially and temporally relevant aspects of real-time signaling.

The results of our simulations show abnormal cellular dynamics at specific locations on arterial geometries; therefore different coupling parameters should be employed at these locations to reflect potentially emergent dysfunctional cellular communication, but there seems to be little evidence to indicate the coupling parameters variation with respect to WSS. This is particularly important, given that simulations presented here span physiological time periods of minutes, while atherosclerotic plaques develop over time scales of months. Modification of localized communication channels as a function of atypical cellular dynamics, such as significantly lower Ca^2+^ concentrations, would allow us to simulate the progression of vascular disease in relatively short simulations.

## Data availability statement

The computational model and required input files for this study can be found in the Coupled Cells Github release: https://github.com/BlueFern/CoupledCells/releases/tag/v-4.0.

## Author contributions

All authors contributed equally to the direction and ideas of the presented work. SD and CZ generated the new surface meshes, extended the physiological model, and ran parallel simulations. SM produced the input ATP maps using computational fluid dynamics methods. SD, CZ, and TD developed the manuscript.

### Conflict of interest statement

The authors declare that the research was conducted in the absence of any commercial or financial relationships that could be construed as a potential conflict of interest.

## Footnotes

^1^https://goo.gl/AQivPz—University of Canterbury High Performance Computing channel on YouTube.
