# Supplementary material for: Coronary Smooth Muscle Cell Calcium Dynamics: Effects of Bifurcation Angle on Atheroprone Conditions
Source: Front Physiol. 2018 Oct 31;9:1528. doi: 10.3389/fphys.2018.01528 (PMC6220094; doi:10.3389/fphys.2018.01528)
Supplement: Supplementary file 1 [file Table_1.PDF]

Supplementary Material: Coupled Cells Mathematical Model

A single pair EC/SMC model from the work of Koenigsberger et al. (2005) accounts for the essential mechanisms of IP_3-induced cytosolic calcium release and the cascade of the triggered processes. The work of Shaikh et al. (2012) builds on the single SMC and EC Koenigsberger model with the homocellular and heterocellular coupling. Similar to the intracellular ion concentrations, the coupling terms are defined as ion fluxes; thus the intercellular fluxes are added to the corresponding conservation equations for the intracellular concentrations. Finally, this study introduced a more physiologically accurate IP_3 pathway based on the work of both Lemon et al. (2003) and Bennett et al. (2005), and electro-diffusion from Jacobsen et al. (2007). The ODEs and fluxes for the resulting single pair EC/SMC model are defined below, while the definitions and values for their parameters can be found in Coupled cells model parameters.

1 EC/SMC ODES

The following equations (S1)–(S5) specify the dynamics for cytosolic calcium concentration (c), SR calcium concentration (s), membrane potential (v), cytosolic IP_3 concentration ($\mathcal{I}$), and the open state probability of the calcium-activated potassium channels (ω) for the SMC layer.

$$\frac{dc}{dt} = \underbrace{J_{IP_3} - J_{SR\_uptake} + J_{CICR} - J_{eff} + J_{leak} - J_{VOCC} + J_{Na/Ca}}_{Cytosolic\ SMC\ Ca^{2+}\ terms} + \underbrace{J_{Ca}^{SMC\leftrightarrow SMC} + J_{Ca}^{SMC\leftrightarrow EC}}_{Coupling\ Ca^{2+}\ terms} \tag{S1}$$

$$\frac{ds}{dt} = \underbrace{J_{SR\_uptake} - J_{CICR} - J_{leak}}_{SR\ SMC\ Ca^{2+}\ terms} \tag{S2}$$

$$\frac{dv}{dt} = \underbrace{\gamma\left(-J_{Na/K} - J_{Cl} - 2J_{VOCC} - J_{Na/Ca} - J_K\right)}_{SMC\ membrane\ potential\ terms} + \frac{1}{C_m}\underbrace{\left(I_{gap}^{SMC\leftrightarrow SMC} + I_{gap}^{SMC\leftrightarrow EC}\right)}_{Gap\ junction\ terms} \tag{S3}$$

$$\frac{d\mathcal{I}}{dt} = \underbrace{-J_{degrade}}_{Cytosolic\ SMC\ IP_3\ term} + \underbrace{J_{IP_3}^{SMC\leftrightarrow SMC} + J_{IP_3}^{SMC\leftrightarrow EC}}_{Coupling\ IP3\ terms} \tag{S4}$$

$$\frac{d\omega}{dt} = \underbrace{\lambda\left(K_{activation} - \omega\right)}_{K\ channel\ term} \tag{S5}$$

The following Eqs (S6)–(S10) specify the dynamics for cytosolic calcium concentration ($\tilde{c}$), SR calcium concentration ($\tilde{s}$), membrane potential ($\tilde{v}$), cytosolic IP$_3$ concentration ($\tilde{\mathcal{I}}$), and activated G protein ($\tilde{G_{prot}}$) for the EC layer.

$$\begin{aligned}\frac{d\tilde{c}}{dt} = & \underbrace{\tilde{J}_{IP_3} - \tilde{J}_{ER\_uptake} + \tilde{J}_{CICR} - \tilde{J}_{eff} + \tilde{J}_{leak} - \tilde{J}_{cation} + \tilde{J}_0}_{Cytosolic\ EC\ Ca^{2+}\ terms} \\ & + \underbrace{\tilde{J}_{Ca}^{EC\leftrightarrow EC} + \tilde{J}_{Ca}^{SMC\leftrightarrow EC}}_{Coupling\ Ca^{2+}\ terms}\end{aligned} \tag{S6}$$

$$\frac{d\tilde{s}}{dt} = \underbrace{\tilde{J}_{ER\_uptake} - \tilde{J}_{CICR} - \tilde{J}_{leak}}_{ER\ EC\ Ca^{2+}\ terms} \tag{S7}$$

$$\frac{d\tilde{v}}{dt} = \frac{1}{\tilde{C}_m}\Bigg(\underbrace{\tilde{I}_K + \tilde{I}_{residual}}_{EC\ V_m\ terms} + \underbrace{\tilde{I}_{gap}^{EC\leftrightarrow EC} + \tilde{I}_{gap}^{SMC\leftrightarrow EC}}_{Gap\ junction\ terms}\Bigg) \tag{S8}$$

$$\frac{d\tilde{\mathcal{I}}}{dt} = \underbrace{\frac{r_h PIP_{2tot} \cdot u_c}{N_a \cdot V_{EC}} - J_{degrade}}_{Cytosolic\ EC\ IP_3\ terms} + \underbrace{J_{IP_3}^{EC\leftrightarrow EC} + J_{IP_3}^{SMC\leftrightarrow EC}}_{Coupling\ IP3\ terms} \tag{S9}$$

$$\frac{d\tilde{G_{prot}}}{dt} = \underbrace{k_a \cdot (\delta + \rho_{P2Y}) \cdot (G_{prot_{tot}} - \tilde{G_{prot}}) - k_d \cdot \tilde{G_{prot}}}_{EC\ G\ protein\ terms}, \tag{S10}$$

2 COUPLING COMPONENTS

The work of Shaikh et al. (2012) provides the following definitions for the homocellular coupling components of the conservation equations for the adjacent SMCs include Ca^{2+} (S11) and IP$_3$ (S12)

concentration fluxes.

$$J_{\text{Ca}}^{\text{SMC}\leftrightarrow\text{SMC}} = -p_{Ca}\left(c - c_k\right) \tag{S11}$$

$$J_{IP_3}^{SMC\leftrightarrow SMC} = -p_{IP_3}\left(\mathcal{I} - \mathcal{I}_k\right) \tag{S12}$$

Similarly, the homocellular coupling components of the conservation equations for the adjacent ECs include Ca^{2+} (S13) and IP_3 (S14) concentration fluxes.

$$J_{Ca}^{EC\leftrightarrow EC} = -\tilde{p}_{Ca}\left(\tilde{c} - \tilde{c}_l\right) \tag{S13}$$

$$J_{IP_3}^{EC\leftrightarrow EC} = -\tilde{p}_{IP_3}\left(\tilde{\mathcal{I}} - \tilde{\mathcal{I}}_l\right) \tag{S14}$$

In the equations above the k and l subscripts are the indices of the adjacent SMCs and ECs respectively.

The work of Shaikh et al. (2012) provides the following definitions for the heterocellular coupling components of the conservation equations for an SMC and its adjacent ECs for Ca^{2+} (S15) and IP_3 (S16).

$$J_{Ca}^{SMC\leftrightarrow EC} = -P_{Ca}\left(c - c_m\right) \tag{S15}$$

$$J_{IP_3}^{SMC\leftrightarrow EC} = -P_{IP_3}\left(\mathcal{I} - \mathcal{I}_m\right) \tag{S16}$$

Similarly, the heterocellular coupling components of the conservation equations for an EC and its adjacent SMCs include Ca^{2+} (S17) and IP_3 (S18) concentration fluxes.

$$J_{Ca}^{SMC\leftrightarrow EC} = -\tilde{P}_{Ca}\left(\tilde{c} - \tilde{c}_n\right) \tag{S17}$$

$$J_{IP_3}^{SMC\leftrightarrow EC} = -\tilde{P}_{IP_3}\left(\tilde{\mathcal{I}} - \tilde{\mathcal{I}}_n\right) \tag{S18}$$

In the equations above the m and n subscripts are the indices of the ECs adjacent to a given SMC and SMCs adjacent to a given EC respectively.

The definition of gap-junction currents provided in the work of Jacobsen et al. (2007) is used to couple both ECs and SMCs, hetero- and homocellularly, with the form:

$$I_{gap,\phi} = P_x\, \sigma\, A_{segment}\, F\left[\nabla[\phi] + \left(\frac{zF}{RT}\,\overline{[\phi]}\,\nabla V_m\right)\right]. \tag{S19}$$

Here $\overline{[\phi]}$ = $([\phi]_a + [\phi]_b)\ /\ 2$, which is the average concentration of ion ϕ between two cells a and b, $\nabla V_m \simeq \frac{V_{m,a} - V_{m,b}}{\delta x}$, which is the membrane potential gradient, and $\nabla[\phi] \simeq \frac{[\phi]_a - [\phi]_b}{\delta x}$.

3 INTRACELLULAR SMC FLUXES

IP_3-induced Ca^{2+} release:

$$J_{\mathrm{IP_3}} = F\frac{\mathcal{I}^2}{K_r^2 + \mathcal{I}^2}. \tag{S20}$$

Ca^{2+}-induced Ca^{2+} release from SR into cytosol:

$$J_{\mathrm{CICR}} = C\frac{s^2}{s_c^2 + s^2}\frac{c^4}{c_c^4 + c^4}. \tag{S21}$$

Ca^{2+} transfer back into the SR via the Ca^{2+}-activated SERCA pump:

$$J_{\mathrm{SR\_uptake}} = B\frac{c^2}{c_b^2 + c^2}. \tag{S22}$$

Ca^{2+} leak from SR:

$$J_{\mathrm{leak}} = Ls. \tag{S23}$$

Influx of extracellular Ca^{2+} via VOCC:

$$J_{\mathrm{VOCC}} = G_{\mathrm{Ca}}\frac{v - v_{\mathrm{Ca_1}}}{1 + \mathrm{e}^{-[(v - v_{\mathrm{Ca_2}})/R_{\mathrm{Ca}}]}}. \tag{S24}$$

Efflux of Ca^{2+} via the Na^+/Ca^{2+} exchanger:

$$J_{\mathrm{Na/Ca}} = G_{\mathrm{Na/Ca}}\frac{c}{c + c_{\mathrm{Na/Ca}}}\left(v - v_{\mathrm{Na/Ca}}\right). \tag{S25}$$

Removal of intracellular Ca^{2+} through the plasma membrane Ca^{2+}-ATPase (PMCA) pump:

$$J_{\mathrm{eff}} = Dc\left(1 + \frac{v - v_d}{R_d}\right). \tag{S26}$$

Function of the Ca^{2+}-activated K^+ channels determined by the cytosolic Ca^{2+} concentration:

$$K_{\mathrm{activation}} = \frac{(c + c_\omega)^2}{(c + c_\omega)^2 + \beta\mathrm{e}^{-\left[(v - v_{Ca_3})/R_K\right]}}. \tag{S27}$$

Potassium efflux through the plasma membrane-bound BK_{Ca} channels, where ω is the open channel probability of the Ca^{2+}-activated channels expressed as a function of $K_{activation}$:

$$J_K = G_{\mathrm{K}}\omega\left(v - v_k\right). \tag{S28}$$

Influx of chloride ions caused by the plasma membrane depolarisation:

$$J_{Cl} = G_{\text{Cl}} \left(v - v_{Cl} \right). \tag{S29}$$

Constant rate efflux of potassium through the Na^{+}/K^{+} pump:

$$J_{\text{Na/K}} = F_{Na/K}. \tag{S30}$$

Linear IP_3 concentration degradation:

$$J_{degrade} = k\mathcal{I}. \tag{S31}$$

4 INTRACELLULAR EC FLUXES

IP_3-induced Ca^{2+} release:

$$\tilde{J}_{\text{IP}_3} = \tilde{F} \frac{\tilde{\mathcal{I}}^2}{\tilde{K}_r^2 + \tilde{\mathcal{I}}^2}. \tag{S32}$$

Ca^{2+}-induced Ca^{2+} release from ER into cytosol:

$$\tilde{J}_{\text{CICR}} = \tilde{C} \frac{\tilde{s}^2}{\tilde{s}_r^2 + \tilde{s}^2} \frac{\tilde{c}^4}{\tilde{c}_c^4 + \tilde{c}^4}. \tag{S33}$$

Ca^{2+} transfer back into the ER via the Ca^{2+}-activated SERCA pump:

$$\tilde{J}_{\text{ER\_uptake}} = \tilde{B} \frac{\tilde{c}^2}{\tilde{c}_b^2 + \tilde{c}^2}. \tag{S34}$$

Ca^{2+} leak from ER:

$$\tilde{J}_{\text{leak}} = \tilde{L}\tilde{s}. \tag{S35}$$

Removal of intracellular Ca^{2+} through the PMCA pump:

$$\tilde{J}_{\text{eff}} = \tilde{D}\tilde{c}. \tag{S36}$$

Calcium influx through non-selective cation channels:

$$\tilde{J}_{\text{cation}} = \tilde{G}_{\text{cat}} \left(E_{\text{Ca}} - \tilde{v} \right) \times \frac{1}{2} \left(1 + \tanh \left(\frac{\log_{10} \tilde{c} - \tilde{m}_{3_{\text{cat}}}}{\tilde{m}_{4_{\text{cat}}}} \right) \right). \tag{S37}$$

Constituent currents determined by the potassium efflux through large and small Ca^{2+}-activated channels:

$$\tilde{I}_{\text{K}} = \tilde{G}_{\text{tot}} \left(\tilde{v} - \tilde{v}_{\text{K}}\right) \left(\tilde{J}_{\text{BK}_{\text{Ca}}} - \tilde{J}_{\text{SK}_{\text{Ca}}}\right), \tag{S38}$$

where

$$\tilde{J}_{\text{BK}_{\text{Ca}}} = \frac{0.4}{2} \left(1 + \tanh\left(\frac{\left(\log_{10}\tilde{c} - \tilde{c}_K\right)\left(\tilde{v} - \tilde{b}_K\right) - \tilde{a}_{K1}}{\tilde{m}_{3b}\left(\tilde{v} + \tilde{a}_{K2}\left(\log_{10}\tilde{c} - \tilde{c}_K\right) - \tilde{b}_K\right)^2 + \tilde{m}_{4\text{b}}}\right)\right) \tag{S39}$$

and

$$\tilde{J}_{\text{SK}_{\text{Ca}}} = \frac{0.6}{2} \left(1 + \tanh\left(\frac{\log_{10}\tilde{c} - \tilde{m}_{3s}}{\tilde{m}_{4s}}\right)\right). \tag{S40}$$

Residual current comprising an inward sodium or potassium current and an outward chloride current:

$$\tilde{I}_{\text{residual}} = \tilde{G}_{\text{R}} \left(\tilde{v} - \tilde{v}_{\text{rest}}\right). \tag{S41}$$

Linear IP_3 concentration degradation:

$$\tilde{J}_{degrade} = \tilde{k}\tilde{\mathcal{I}}. \tag{S42}$$

Ratio of bound to total P2Y receptors:

$$\rho_{P2Y} = \frac{[ATP]}{K_{ATP} + [ATP]}. \tag{S43}$$

Hydrolysis rate of PIP_2:

$$r_h = \alpha \left(\frac{\tilde{c}}{\tilde{c} + K_{Ca}}\right) \tilde{G_{prot}}. \tag{S44}$$

5 COUPLED CELLS MODEL PARAMETERS

Table S1 and Table S2 provide the parameters for a single SMC and EC used in the model of Koenigsberger et al. (2005). Table S3 provides the parameters for the IP_3 pathway model (Lemon et al., 2003; Bennett et al., 2005), and Table S4 provides the parameters used in the gap-junction currents equation (Jacobsen et al., 2007).

Parameter	Definition	Value
F	Maximal rate of activation-dependent calcium influx	0.23 μM s^{-1}
K_r	Half saturation constant for agonist-dependent calcium entry	1 μM
G_{Ca}	Whole cell conductance for VOCCs	0.00129 μM mV^{-1} s^{-1}
v_{Ca1}	Reversal potential for VOCCs	100 mV
v_{Ca2}	Half-point of the VOCC activation sigmoidal	-24 mV
R_{Ca}	Maximum slope of the VOCC activation sigmoidal	8.5 mV
$G_{Na/Ca}$	Whole cell conductance for Na^+/Ca^{2+} exchange	0.00316 μM mV^{-1} s^{-1}
$c_{Na/Ca}$	Half-point for activation of Na^+/Ca^{2+} exchange by Ca^{2+}	0.5 μM
$v_{Na/Ca}$	Reversal potential for the Na^+/Ca^{2+} exchanger	-30 mV
B	SR uptake rate constant	2.025 μM s^{-1}
c_b	Half-point of the SERCA activation sigmoidal	1.0 μM
C	CICR rate constant	55 μM s^{-1}
s_c	Half-point of the CICR Ca^{2+} efflux sigmoidal	2.0 μM
c_c	Half-point of the CICR activation sigmoidal	0.9 μM
D	Rate constant for Ca^{2+} extrusion by the ATPase pump	0.24 s^{-1}
v_d	Intercept of voltage dependence of extrusion ATPase	-100 mV
R_d	Slope of voltage dependence of extrusion ATPase	250 mV
L	Leak from SR rate constant	0.025 s^{-1}
γ	Scaling factor relating net movement of ion fluxes to the membrane potential (inversely related to cell capacitance)	1970 mV^{-1} μM
$F_{Na/K}$	Net whole cell flux via the Na^+–K^+–ATPase	0.0432 μM s^{-1}
G_{Cl}	Whole cell conductance for Cl^- current	0.00134 μM mV^{-1} s^{-1}
v_{Cl}	Reversal potential for Cl^- channels	-25 mV
G_K	Whole cell conductance for K^+ efflux	0.00446 μM mV^{-1} s^{-1}
v_K	Reversal potential for K^+	-94 mV
λ	Rate constant for net K_{Ca} channel opening	45
c_ω	Translation factor for Ca^{2+} dependence of K_{Ca} channel activation sigmoidal	0 μM
β	Translation factor for membrane potential dependence of K_{Ca} channel activation sigmoidal	0.13 μM^2
v_{Ca3}	Half-point for the K_{Ca} channel activation sigmoidal	-27 mV
R_K	Maximum slope of the K_{Ca} activation sigmoidal	12.0 mV
k	Rate constant of IP_3 degradation	0.1 s^{-1}
p_{Ca}	Homocellular Ca^{2+} coupling coefficient	0.05 s^{-1}
P_{Ca}	Heterocellular Ca^{2+} coupling coefficient	0.0 s^{-1}
p_{IP_3}	Homocellular IP_3 coupling coefficient	0.05 s^{-1}
P_{IP_3}	Heterocellular IP_3 coupling coefficient	0.05 s^{-1}

Table S1. SMC model parameters as described by Koenigsberger et al. (2005).

Parameter	Definition	Value
$\tilde{F}$	Maximal rate of activation-dependent calcium influx	0.23 μM s^{-1}
$\tilde{K}_r$	Half saturation constant for agonist-dependent calcium entry	1 μM
$\tilde{B}$	ER uptake rate constant	0.5 μM s^{-1}
$\tilde{c}_b$	Half-point of the SERCA activation sigmoidal	1.0 μM
$\tilde{C}$	CICR rate constant	5 μM s^{-1}
$\tilde{s}_c$	Half-point of the CICR Ca^{2+} efflux sigmoidal	2.0 μM
$\tilde{c}_c$	Half-point of the CICR activation sigmoidal	0.9 μM
$\tilde{D}$	Rate constant for Ca^{2+} extrusion by the ATPase pump	0.24 s^{-1}
$\tilde{L}$	Leak from SR rate constant	0.025 s^{-1}
$\tilde{k}$	Rate constant of IP_3 degradation	0.1 s^{-1}
$\tilde{G}_{cat}$	Whole cell cation channel conductivity	0.66 $\mu M\ mV^{-1}$ s^{-1}
$\tilde{E}_{Ca}$	Ca^{2+} equilibrium potential	50 μV
$\tilde{m}_{3cat}$	Model constant	-0.18 μM
$\tilde{m}_{4cat}$	Model constant	0.37 μM
$\tilde{J}_0$	Constant calcium influx	0.029 μM s^{-1}
$\tilde{C}_m$	Membrane capacitance	25.8 pF
$\tilde{G}_{tot}$	Total potassium channel conductivity	6927 pS
$\tilde{v}_K$	K^+ equilibrium potential	-80 mV
$\tilde{a}_{K1}$	Model constant	53.3 $\mu M\ mV$
$\tilde{a}_{K2}$	Model constant	53.3 $mV\ \mu M^{-1}$
$\tilde{b}_K$	Model constant	-80.8 mV
$\tilde{c}_K$	Model constant	-0.4 mM
$\tilde{m}_{3b}$	Model constant	1.32×10^3 mV^{-1}
$\tilde{m}_{4b}$	Model constant	0.30 $\mu M\ mV$
$\tilde{m}_{3s}$	Model constant	-0.28 μM
$\tilde{m}_{4s}$	Model constant	0.389 μM
$\tilde{G}_R$	Residual current conductivity	955 pS
$\tilde{v}_{rest}$	Membrane resting potential	-31.1 mV
$\tilde{p}_{Ca}$	Homocellular Ca^{2+} coupling coefficient	0.05 s^{-1}
$\tilde{P}_{Ca}$	Heterocellular Ca^{2+} coupling coefficient	0.0 s^{-1}
$\tilde{p}_{IP_3}$	Homocellular IP_3 coupling coefficient	0.0 s^{-1}
$\tilde{P}_{IP_3}$	Heterocellular IP_3 coupling coefficient	0.05 s^{-1}

Table S2. EC model parameters as described by Koenigsberger et al. (2005).

Parameter	Definition	Value
δ	G protein activity parameter	$1.234 \cdot 10^{-3}$
K_a	G protein activation rate	0.017 s^{-1}
K_d	G protein deactivation rate	0.15 s^{-1}
N_a	Avogadro's constant	$6.02252 \cdot 10^{23} mol^{-1}$
u_c	Convert $mol/\mu m$ into μM	10^{21}
α	Effective signal gain parameter	$2.781 \cdot 10^{-5} s^{-1}$
K_{Ca}	Dissociation Constant	0.4 μM
r_r	Rate of PIP_2 replenishment	10 s^{-1}
$PIP_{2_{tot}}$	Total PIP_2 molecules	$5.0 \cdot 10^7$
$G_{prot_{tot}}$	Total G protein molecules	$1.0 \cdot 10^5$
k_{deg}	IP_3 degradation rate	1.25 s^{-1}
K_{ATP}	Michaelis-Menten constant for ATP binding to a P2Y receptor	2.0 μM

Table S3. Model parameter values used in the IP_3 pathway from Lemon et al. (2003).

Parameter	Definition	Value
$A_{segment}$	Averaged area of cells in contact	Varies on the cells in contact
$P_{general}$	Standard value of permeability	5×10^{-8} $m/s.$
F	Faraday Constant	96487.0 C/mol
R	Universal Gas Constant	8314.0 $mJ/molK$
T	Absolute temperature	293.0 K
σ	Fraction of circumference directed toward neighbouring cell	0.15 for homocellular coupling, 0.05 for heterocellular coupling

Table S4. Gap junction current parameter values taken from Jacobsen et al. (2007).

Parameter	Definition	Value
$[Na^+]_{cyt,smc}$	Intracellular concentration of Sodium in SMCs	8.4 mM
$[K^+]_{cyt,smc}$	Intracellular concentration of Potassium in SMCs	140.0 mM
$[Cl^-]_{cyt,smc}$	Intracellular concentration of Chloride in SMCs	59.4 mM
$[Na^+]_{cyt,ec}$	Intracellular concentration of Sodium in ECs	8.3 mM
$[K^+]_{cyt,ec}$	Intracellular concentration of Potassium in ECs	128.0 mM
$[Cl^-]_{cyt,ec}$	Intracellular concentration of Chloride in ECs	87.0 mM

Table S5. EC and SMC intracellular concentrations of Na^+, K^+, and Cl^- taken from Kapela et al. (2008).

Parameter	Definition	Value
v_p	Maximum rate of phosphatase	5 $\mu M s^{-1}$
V_{mk}	Maximum rate of the kinase at saturation	40 $\mu M s^{-1}$
K_a	Constant of activation of the kinase by cytosolic Ca^{2+}	2.5 μM
W_T	Total amount of protein substrate	1 μM
K_1	Michaelis constants of kinase	0.1
K_2	Michaelis constants of phosphatase	0.1

Table S6. Simulation parameters for protein phosphorylation from Goldbeter et al. (1990).

REFERENCES

Bennett, M. R., Farnell, L., and Gibson, W. (2005). A Quantitative Model of Purinergic Junctional Transmission of Calcium Waves in Astrocyte Networks. *Biophysical Journal* 89, 2235–2250. doi:10.1529/biophysj.105.062968

Goldbeter, A., Dupont, G., and Berridge, M. J. (1990). Minimal model for signal-induced Ca^{2+} oscillations and for their frequency encoding through protein phosphorylation. *Proceedings of the National Academy of Sciences* 87, 1461–1465

Jacobsen, J. C. B., Aalkjaer, C., Nilsson, H., Matchkov, V. V., Freiberg, J., and Holstein-Rathlou, N.-H. (2007). A model of smooth muscle cell synchronization in the arterial wall. *American Journal of Physiology - Heart and Circulatory Physiology* 293, H229–H237. doi:10.1152/ajpheart.00727.2006

Kapela, A., Bezerianos, A., and Tsoukias, N. M. (2008). A mathematical model of Ca2+ dynamics in rat mesenteric smooth muscle cell: Agonist and NO stimulation. *Journal of Theoretical Biology* 253, 238–260. doi:10.1016/j.jtbi.2008.03.004

Koenigsberger, M., Sauser, R., Beny, J.-L., Meister, J.-J., Bény, J.-L., and Meister, J.-J. (2005). Role of the endothelium on arterial vasomotion. *Biophysical Journal* 88, 3845–3854. doi:10.1529/biophysj.104.054965

Lemon, G., Gibson, W. G., and Bennett, M. R. (2003). Metabotropic receptor activation, desensitization and sequestration - I: Modelling calcium and inositol 1,4,5-trisphosphate dynamics following receptor activation. *Journal of Theoretical Biology* 223, 93–111. doi:10.1016/S0022-5193(03)00079-1

Shaikh, M. A., Wall, D. J. N., and David, T. (2012). Macro-scale phenomena of arterial coupled cells: a massively parallel simulation. *Journal of The Royal Society Interface* 9, 972–987. doi:10.1098/rsif.2011.0453
